# The College Course and Professional Training

**Published:** 1902-03

**Authors:** S. M. Macvane

**Affiliations:** Boston, Mass.


### THE COLLEGE COURSE AND PROFESSIONAL TRAINING.[1]

BY S. M. MACVANE, PH.D., BOSTON, MASS.[2]

Dr. Pond and Gentlemen of the Dental Academy,—I can say, sincerely, it is a great pleasure to sit for a while between dentists, and get one of your number in the chair at last, while I do the operating. If anything I say shall prove distasteful to any or all of you, I shall have, remembering the past, certain grounds for satisfaction.

I wish to speak a little this evening about a subject that has interested another set of us very much, and I assume interests you also, and that is, some points in the relations between professional and liberal education, as we are wont to call it. I do not know whether you call college education distinctly liberal education or not. If you do not, please excuse my using the word. I suppose all education is liberal in a way, but there are degrees of liberality and degrees of professionalism.

The question of professional education, I may say at once, is a new one in the world, new in America, and new everywhere. It has brought some very troublesome questions between what are

---

[1] Read before the thirty-fourth annual meeting and banquet of the American Academy of Dental Science, Boston, November 13, 1901.

[2] McLean Professor of Ancient and Modern History in Harvard University.

called the colleges and what are called the professional schools. There is a form of it that is particularly American. Other countries have no colleges such as we have. No other country attempts to keep a grade between what we call secondary schools and the university. We got our colleges in a perfectly natural way, and have been developing them, building on them, and strengthening them all around, until they have become very important parts of our civilization.

Now, I say professional education is new. You all know, I suppose, that in your own profession fifty years back there was hardly any provision for the education of men for the dental profession. I believe the first dental college in the world was the one established in Baltimore in 1839. And law schools are comparatively recent. Of course, theological schools are not. But the professions of law, of medicine, and of dentistry are certainly modern professions, and the provisions for training men in them is quite recent. For the most part, in this country provision for professional training came later than the Civil War.

Now, it so happens that the period since the Civil War has also been a period of the development of the colleges. The colleges are some of them quite old, of course. Harvard boasts its age, and so does Yale; but there has been more change, in Harvard at all events, since the Civil War than in the two centuries and a half before. That is, there was formerly a stereotyped, old-fashioned college training, prescribed courses, carried on in a certain way; once in a while they would add something, and once in a while change something, but the general characteristics remained the same. But in the last half-century the world has waked up, and the colleges have waked up also. The fact has become recognized that science is an advancing thing, and life is a very multifarious thing, and every educated man ought to know well about some phase or feature of ourselves or our surroundings. We think we have learned that you cannot get everybody to learn a little dab of everything, without preventing everybody from learning anything very well. You have got to choose somewhere: the question is, How far will you let one choose? If I may speak of Harvard more than once, I will say that Harvard has taken the risk of going the whole length, and saying to young men that they may choose their studies for themselves; taking care, of course, not to offer anything that is not a reasonably good study. We think

Harvard is not offering some poor and some good studies, but all good. We place them all on equal terms. The result has been that we think we are getting much better work done than was ever done before, much more real studying done. We are training men to study; not to get up a lesson for somebody overnight, and recite it the next morning, but to go to work on a study, and use books and teachers as instruments.

Then, of course, on the scientific side there has been an enormous development. Colleges provide not only the scientific departments, but all the colleges are now run on a scientific basis. Taking my own department of history, there is no comparison between the way our work is done by our more advanced students in history now in Harvard and the way it was done fifty years ago. Now, any set of men who expect to master history at all must develop a spirit of going to the sources of history and working for themselves, must learn to like that kind of work and pursue it because they love it. We think that is the way to train men.

Of course, in the scientific departments, strictly, as you know better than I do, there has been an enormous development of the the spirit of research. The same thing we try to develop in history is developed in science. A great outfit of teachers and apparatus is required for this, and the colleges are undertaking to provide these, to keep up the lights of learning, and to promote original research for its own value; not because it may help anybody in business, but because it will help everybody to learn something about the world we live in. Those things we think are good in themselves.

I will say I think no college has found four years too long a time to spend in training young men. In this new spirit, and with these new educational outlooks and methods, four years are a very short time to spend. Now, while the colleges have been waking up to do better work, and therefore are inclined to ask for more time, and not less time, from the young men, the professional schools have waked up too. Professional spirit has waked up to the fact that professional work too may be carried on in a new spirit. Theology to-day is a very different sort of study from what it was fifty years ago. Law has enormously changed. You do not any more go to a law school to hear a course or two of lectures, and then pass an examination, and go into practice. Students now in all the reputable law schools have to make a long, arduous course of study of the raw material, the cases. Instead of manufactured text-books, they go to the opinions of the judges, where they have to go eventually in any event if they are ever going to be real lawyers.

Then in your scientific departments, you know better than I do what is going on with microscopes and all that, and how much time it takes. When you bring your student face to face with the human frame, and the evidences and causes of disease, you have a long task on your hands. We in the liberal sciences think we have made discoveries and we have something to give to young people that is worth detaining them for; and you professional men think that your professional training now requires to be long, and we had better let them come early to you. That is the origin, I suppose, of the demand for what is called the three years' course, and that is what I want to talk to you about particularly; and if, in doing so, I seem to show my teeth as a college man, please excuse me; you will understand it.

I am not one of those who believe that the college course ought to be reduced to three years. I will be frank with you at the outset, and if you do not like what I say, you will pardon me, at all events. It has been my settled opinion that the American public as a whole, the college-patronizing public, has not shown, thus far, any strong desire, any general desire, for the reduction of the college course to three years. The demand for it, so far as I have seen, has come from some of the professional schools, or from men who tell you on abstract principles that it is unsuitable to give four years for a liberal training, between the preparatory schools on one side and the professional schools on the other.

It is my opinion that the American public, the better part of it, want the best thing that they can get. They do not want a second-rate article in this country. One finds evidences of that in places where you very little expect it. In riding on our railroads one sees it: we have not our first, second, and third class in America. If you put on first, second, and third classes, I do not know which class our people would use, but I feel sure they would mostly ride in the same class. You will not find ordinary people voluntarily submitting to a poorer article, at least in public, than they know their richer neighbors are getting. There is a great deal of pride of equality, democratic equality, in America, which is at bottom a good thing. Anyway, it is there, and the form it takes in this matter of education, it seems to me, is that your ordinary intelligent American, sending his boy to college, and embarking him on a professional or other training, wants to get the best he can afford; and we have a very large class, larger than any other country, who can afford the best there is. I see evidences of that in relation to the colleges, in the fact that the colleges that have made the largest demands in the way of entrance requirements, and have a four years' course, have grown more rapidly than those that have offered easy terms of admission or a shorter course to get through.

Let me give just one example, Johns Hopkins University; you have all heard of it, and the world has heard of it. It is a very notable institution. Johns Hopkins University has offered always a three years' course for the A.B. degree; and it is a well-known fact that its entrance requirements are no higher than those of Harvard or Yale. But the Johns Hopkins undergraduate department does not grow. It has only about one hundred and thirty candidates all told. Harvard sometimes grows that much in one year. There was a time when we were more particular about a four years' course at Harvard than, I am sorry to say, we are now. I have never yet known a college that had a four years' course cutting it frankly down to three, but I have known of a three years' course frankly raised to four; which does not look as if there was a large public demand for a three years' course: it points the other way. However, it may be assumed that there are individual parents who think it would be better if all young men went through a three-year college course only; they would be willing to have their sons do that if others did it. But they do not choose a three-year college, though the choice is quite open to them.

On the other hand, while it is the fashion to have a four years' course, there have been some changes made in American colleges with a view to meet the case of men who cannot afford, or are supposed not to be able to afford, first, the long preliminary training required to get into a high-grade college, and then the four years' course. Unfortunately, a large proportion of our colleges have entrance requirements that look well on paper, but they admit without examining on these, taking certificates from teachers, etc., as evidence that men have gone over the ground. Then there are others that have a high-sounding set of requirements, with examinations, but do not insist on a very substantial performance.

We all admit with some deficiencies, but all colleges should have real examinations and insist that before passing candidates shall show some real knowledge.

Then, some of these same colleges have tried experiments in multiplying degrees. There is the degree of Bachelor of Letters, and the degree of Bachelor of Philosophy, invented as cheap substitutes, I should say, for the A.B. degree. The colleges that have those have the Bachelor of Arts degree besides, and require a four years' course for that, and also, nominally, require a pretty high admission outfit, and then have these side-shows of Bachelor of Philosophy and Bachelor of Letters. Those degrees do, it must be said, attract a considerable proportion of the students in the colleges that have them. They are probably meeting a need of some sort among the patrons of those institutions, but the larger universities, Harvard and Yale at all events, do not offer any such degrees at all; and they have apparently clearly resolved that, whatever solution of the education question they shall take, it shall not be that solution of introducing cheap substitutes for the Bachelor of Arts degree, but something done for the arts degree itself.

Some have a simple solution, and that is simply to cut off one year from the arts course, make it three frankly, and be done. Now, that remedy goes hard with the college faculty men, even with those who think it the least objectionable method. They have been working for half a century, nearly, improving the standards and the methods of the colleges, and were getting the colleges to a position where they were doing, we thought, really good work; at all events, had begun to do really good work. And now comes a proposition to spoil it all, or spoil the best part of it, by striking out the last year. The last year of a student's years in college has always been the best year, of course. Young men do not get the proper degree of maturity for real university work until they get to be twenty-one years of age, or so. I feel that young men, without much additional effort, accomplish vastly more in the senior year than in any other year of the course. They are prepared to do better work. The proposition now comes to strike off the senior year, and let all that go. College professors would be more than human, I think, if they did not feel sorry to have that happen: even those who are willing to cut it off, in supposed answer to public demand, will tell you they are sorry,—sorry they could not leave a good thing alone.

There is another plan which is in use at Harvard for meeting this difficulty, and apparently suits many of the advocates of the reduction, and that is to throw the elements of the four years' course into hodge-podge, abolishing your notions of freshman, sophomore, junior, and senior, each with its prescribed number of courses, and letting the students take the whole dose in three instalments instead of four, doing extra work enough each year to make it count for the fourth year. I do not myself think that method is satisfactory, on the whole. Some of those who favor the reduction in this form, who are voting for it and supporting it, do not like it; and I will tell you one reason, and a strong reason, why. Most of us are pretty clear that the so-called *extra* work is not really extra, in many cases: it would not be well if it were. The best students in Harvard, since I have known it at all events, did enough work already. The fact was that many of them did too much for their own good later: they spent life force in study that ought to have been kept in reserve or used in recreation of some sort. Those men are not really doing extra work; they are merely distributing their work in a different way. They are getting less out of each course than they used to do when they did only four courses a year instead of the five or six they do now. But they get through, and if they pass with sufficiently high grades, they are entitled to the degree at the end of three years. If they pass with but low grades, the faculty has consented to count the work done eventually, not giving the degree at the end of the three years, but giving it to them with their class, allowing them to be absent from the college during the fourth year. That is not an ideal arrangement, and it gives rise to some very embarrassing subordinate questions. Under that arrangement some forty of our students are now in the professional schools, who are excused from the senior year in college, because they already have passed enough courses to make up the stint for the degree.

Those of us who do not think the three years' course really called for are rather unhappy about that, because we think it is a sort of double injury, shortening the course, and spoiling the course; spoiling the chance to get the best work out of the men who have always been our scholars. An ordinary man cannot do scholarly work under these circumstances; it is simply out of the question. Of course, he is helped somewhat by the fact that in the choice of studies he can choose those which co-operate together.

Several courses in history, for instance, may help each other, and so relieve him somewhat; but every course has an independent line of work, after all, if it is well done; and if it is done as it ought to be, it takes time. And those of us who do not like these things are not happy. I confess that I am myself not easy about that phase of our college situation, but I am waiting to see how it works out.

The Board of Overseers (I do not know whether this is telling tales out of school) asked us about two years ago to put into the catalogue a clear and simple statement of the conditions on which men can get the degree of Bachelor of Arts. The faculty took that up and worked over it, and they appointed a committee on it, and the committee reported on it; the faculty worked over that report, and did not much like it. Then various substitutes were offered, and we labored over them, and spent two years over the matter. Only at the last meeting but one we finally adopted something, by a very small majority, as a statement of the ways in which men may get the Bachelor of Arts degree. One difficulty was the question whether it is still a fact with us that ordinarily four years are required,—that there is a sort of presumption in favor of four years, and that the three years' course is more or less experimental. Of course, one set of men wished to put the three years' degree on the same terms as the four; and another set wished to stand for a four years' requirement as the ordinary course, and the three years' course as an exception.

That question of policy meets us at every turn, in our discussion of our college government and courses of study, and all that: we run up against that three years' question every time. The faculty is very much divided about it, and probably Harvard will have to do some drifting as regards that question, and wait for further light.

Meanwhile, you professional men, I suppose, would be, on the whole, inclined to the proposition that professional education is the first thing to be considered; that what we call liberal education must take its chances, or must more or less take its chances, and that the college ought to be satisfied with three years. Perhaps I am doing you uncalled-for service in assuming that, but I have found that that is, on the whole, apt to be the professional state of mind. I judge your chairman, perhaps, would not go so far as that, from some things he dropped in the course of his excellent speech; but I know that a good many professional men do take that position, and take it strongly.

Has it occurred to you that there is a possible danger of overdoing professional school work? I am not asking that you go back to the old days when the barber or the blacksmith was the dentist, and when the apothecary was the physician. Those things have had their day. Remember that only in recent times has the world begun to insist upon strictly professional training to any large extent as a university function. Looking at the evidence of the centuries behind us, we can see that liberal training is by the verdict of time the most important thing. The world had that, and valued it, and clung to it, long before it had the dental colleges or medical schools to any extent. Is it not evidently of more importance to have well-trained men, citizens with trained minds, than to have the younger members of the professions very highly trained at the very moment of their entrance into the profession? Is not your young dentist bound, in any case, to be more or less of a journeyman for some years? Is not the young lawyer bound to be young intellectually? In other words, has he not still to go through a large amount of professional education, professional training, before he shall be a really accomplished doctor of dental medicine, or a really able lawyer? I am sure that it is possible, easily possible, to overrate the importance of keeping a young man a large number of years in the school stage of his profession. A law graduate cannot know all the law; even if the law course be made four years, he is not going to know all the law and be an oracle the moment he opens his office. He will have to take hold of his case, when he gets one, and look up the cases bearing on it; and the best the law school can give him is the ability and the power to get hold of the previous cases of the same general sort, and discretion to select those that bear on his point. If he has that ability trained into him by his course in the law, I think he has got the best thing the law school can give him. It is impossible to fortify him with enough of the law at every point for each case as it comes. It is a question whether two years of training in methods of study of law would not be enough. If a young lawyer were to spend the next two years butting against practice, doing what he could with it, helping and observing older lawyers, and getting the general bearings of his profession, it is a question if that would not be better than spending a third or a fourth year in the law school.

I imagine that other professions are very much in the same case; that it is possible to overrate the value of the third year or the fourth year in university methods of study of the elements of the profession. Of course, now, I speak with diffidence about that, whether I seem to or not. I am aware that in the study of medicine, and probably in your own profession, the new discussions, the new scientific treatment of medical pathology, or whatever you call it, may alter the case. There you need laboratories, I suppose, a very expensive outfit which the individual practitioner could not provide himself with. It may be different in your case. A lawyer, I feel sure, is on a somewhat different basis.

I said some of the colleges are trying to compress four years' work into three years' time, in order to accommodate themselves to a supposed demand for a shortening of the college course. There are other ways in which much has been done that ought to appease this alleged demand. One way is that of offering elective courses that contribute very helpfully towards the study of the professions later, enabling the student who takes these courses to shorten, or make more effective, his professional course. Doctors, for instance, I suppose might use courses in chemistry and biology and kindred work in Harvard, so as to shorten their medical course, or, at all events, to make it more valuable to them, immensely more valuable, I should think. The elective system, besides its other merits, is a very real contribution to professional education.

There is a third way that is tried by some colleges, notably by Columbia University,—the principle of letting candidates for the Bachelor of Arts degree take their fourth year in a professional school, and have that year count towards their Bachelor of Arts degree. Columbia seniors go off into law, or medicine, or any other of the professional schools, and receive, at the end of the year, their Bachelor of Arts degree. That has been discussed frequently at Harvard as a practical solution of the question, but never has found favor with a majority of the faculty. There are various reasons why: one is that the Harvard faculty of arts and sciences would not like to seem to coddle our own professional schools in that way. If a senior might go off to the Harvard Medical School and take his year there, we should have to say that he might also go to Columbia or to Yale. We have a new spirit of courtesy towards the universities that would make it impossible for Harvard,

I think, now to say, "We would let you stay a year in our own medical school, and count it towards your degree, but if you want to go to Yale or Columbia, we cannot count work done there." That would not be at all in keeping with our practice. If we set out to let our students go to any professional school to make the senior year, you can imagine what a set of administrative questions there would be about that at the end of the year. The difficulties we should have in administering the plan would be endless.

We run up against the wall in this question at a great many points in every solution of it that has been suggested, except the frank one of cutting off a year. I am free to say that, so far as I am concerned,—and I think I represent a good many of the other opponents of the three years' course,—I would rather cut off the fourth year altogether, simply let it go, than attempt to work substitutes and contrivances about it, keeping up a pretence of having it when we had ceased to have it. I think that would be the best solution in the long run,—I mean the least objectionable.

But it remains to be seen whether the country wants that or not. All the evidences, so far as I have seen them, point the other way. This country wants a good thing: they want good university training. They do not forget, and do not want us to forget, that not all the students go to professional schools. Are we going to cut down the four years' course to three for all the students, because some of them study medicine later, or law, and so on? Or shall we say to everybody, This country has had a four years' course in its colleges, has shown its ability to support that, has shown a pride in it, and a desire to strengthen it by showering new resources on the colleges all around; and shall we not keep this highly prized national possession intact?

The reasons given for cutting down to three years would all be quite as rational and in place by and by to cut down to two. There really would be no limit, once you begin cutting away the college work. The secondary schools are growing, and quite a large proportion of them would be able to put their students into the sophomore year without exertion, and they may eventually absorb the freshman year. And if we lose the freshman year to the preparatory schools, and lose the senior year to the professional schools, where is the college going to stand? It comes back to what I have said about other countries that have had highly developed secondary schools. They send men from their Latin schools to the professional and university schools: there is no college there. Do we want colleges in America as a part of our outfit, or do we not? That is the real question. Is there room for the college in the American education? Probably we all think there is. But if we really think that, we must try to accommodate other things to what is necessary to the real life of the colleges.

It may be that colleges here could be kept up for a long time on the three years' basis. They could undoubtedly do good work, if not the best work. A young man who gets a three years' degree now, with the improved fitting-school training, is certainly as good a scholar as his grandfather was who took the four years' course. But are we always to be satisfied with what our grandfathers had? We certainly are not so easily satisfied in other things. You are not so minded in the professional line. And is the college to be the only institution that must go back? You will hardly expect a college man to answer "yes."

The practical question is, it seems to me, Is the wealth of America enough to provide a better education than any other country has? I think it is. I think America is willing to do it. And the people who are trying to convince our young men that four years are too many to spend in college work are really doing us a doubtful service. The reduced college course they advocate would doubtless be still a good thing; but it would not be the best thing of which we are capable. When the colleges that set the highest standard for the A.B. degree begin to be abandoned in favor of those that make the least amends, the time will have come for lowering the requirements. But that time gives, as yet, no sign of its approach.

There is one other point that I wish to suggest. In looking over a book this afternoon on professional education, a book issued by the University of New York, I was surprised to discover how few professional schools in this country have admission requirements that amount to anything. There are but two law schools that require for admission any sort of degree, or, indeed, any appreciable college work. Of your own dental schools (there are fifty-six in this country) three have no express admission requirements; eighteen have purely grammar school subjects for entrance; another eighteen seem to require about a first year in the high school course, just the beginnings of French and Latin; eleven others have requirements that would be covered by about two years of the ordinary high school courses; and six of the best may be fairly said to require about a three years' high school course. None of them requires as much as would be required for admission to any college.

As for medical schools, there are, according to the same authority, only two (Harvard and Johns Hopkins) that even pretend to require a degree for entrance. Twenty-nine out of one hundred and fifty-six medical schools simply require grammar school subjects. The rest demand varying quantities of high school work. As a class they make demands for admission far below the admission requirements of the colleges.

The theological schools are above the other professional schools in the way of requirements. More than half of them require some college training,—in most cases the full college course.

What sticks in the crop of some college professors is that the professional schools, without requiring a degree at all, or, indeed, anything that counts for a degree, should undertake to tell the colleges, You ought to lower your degree, so that *your* men may come to us earlier. Why cannot the professional schools, if they are not prepared to demand a degree, demand at least some part of the work that goes towards a degree? Why not put the requirement up to include the work of the junior year, as the Harvard Law School did for a while, or even the work of the sophomore year? Men could be admitted to the professional school on the basis of college record up to any specified point in the course quite as easily as they can be admitted on the strength of a degree. And any step in this direction would throw the influence of the professional school in favor of some degree of liberal education, an influence that has hitherto been lamentably lacking.

